# Climate Change–Based Art and Philosophy Intervention and Mental Health in Children

**DOI:** 10.1001/jamanetworkopen.2025.31298

**Published:** 2025-09-11

**Authors:** Catherine Malboeuf-Hurtubise, Terra Léger-Goodes, Heena Dave, Leigh Hoath, David Lefrançois, Marc-André Éthier, Jonathan Smith, Claudine Fillion, Emilie McLean, Zachary Fry, Kevin Péloquin, Catherine M. Herba

**Affiliations:** 1 Bishop’s University, Sherbrooke, Quebec, Canada; 2 Centre Hospitalier Universitaire de Sherbrooke (CHUS) Research Center, Sherbrooke, Quebec, Canada; 3 Université du Québec à Montréal, Montréal, Quebec, Canada; 4 University of Stirling, Stirling, United Kingdom; 5 Leeds Trinity University, Leeds, United Kingdom; 6 Université du Québec en Outaouais, Saint-Jérôme, Quebec, Canada; 7 Centre de recherche interuniversitaire sur la formation et la profession enseignante, Montreal, Quebec, Canada; 8 Université de Montréal, Montréal, Quebec, Canada; 9 Université de Sherbrooke, Sherbrooke, Quebec, Canada; 10 Centre Hospitalier Universitaire Sainte-Justine Azrieli Research Center, Montréal, Quebec, Canada

## Abstract

This comparative effectiveness study compares climate change–related mental health outcomes among elementary school students receiving arts-based interventions and arts-and-philosophy–based interventions.

## Introduction

Children are becoming increasingly aware of the accelerating^1^ climate crisis and more vulnerable to developing eco-anxiety,^2^ an emotional reaction that arises when an individual recognizes the impact humans have on the environment (see eAppendix in Supplement 1).^3^ A promising approach for children to discuss their eco-anxiety in schools is through the integration of artistic creation and philosophical inquiry.^3^This study compared the outcomes of arts-based interventions (ABI) and arts-and-philosophy–based interventions (APBI), centered on the theme of climate change, with elementary school students’ eco-anxiety as a primary outcome, and their intolerance to distress, hope, and mental health as secondary outcomes.

## Methods

This comparative effectiveness study was implemented to compare the before-and-after outcomes of both ABI and APBI. Mixed analyses of variance were used to compare preintervention and postintervention scores between and within groups. The Bonferroni-Holm correction method was applied, with a significance threshold of *P* < .01, hypotheses were tested using mixed model analyses of variance within SPSS v. 29. Effect sizes were computed using partial η^2^ values (eMethods in Supplement 1). The study obtained ethics approval from the Bishop’s University Research and Ethics Board. Parents’ written informed consent and children’s verbal assent were obtained. This manuscript follows the ISPOR reporting guidelines.

## Results

A total of 238 students (129 female [54.1%];  mean [SD] age,  9.76 [1.09] years; age range, 7-12 years) participated in the study. Results revealed significant preintervention vs postintervention differences for global eco-anxiety in both groups (mean [SD] preintervention vs postintervention scores, 0.98 [0.62] vs 1.27 [0.70] for ABI and 0.97 [0.59] vs 1.07 [0.63] for APBI; *P* = .006; partial η^2^ = 0.03) (Tables 1 and 2). While the interaction was a trend, sensitivity analyses suggested scores in the ABI group were more pronounced. Both groups showed significant increases in their eco-anxiety regarding their personal impact on the planet (mean [SD] preintervention vs postintervention scores, 1.50 [1.44] vs 3.02 [0.86] for ABI and 1.24 [1.16] vs 2.81 [0.87] for APBI; *P* < .001; partial η^2^ = 0.552) and decreases in rumination (mean [SD] preintervention vs postintervention scores, 1.22 [0.99] vs 1.19 [1.22] for ABI and 0.95 [0.94] vs 0.67 [0.99] for APBI; *P* = .01; partial η^2^ = 0.029).

**Table 1.** Preintervention vs Postintervention Scores for APBI vs ABI

Measure	Mean (SD) scores			
	APBI (n = 169)		ABI (n = 69)	
	Preintervention	Postintervention	Preintervention	Postintervention
Age, y	9.84 (0.98)	NA	9.43 (1.3)	NA
Primary outcome: Eco-anxiety				
Global	0.97 (0.59)	1.07 (0.63)	0.98 (0.62)	1.27 (0.70)
Rumination	0.95 (0.94)	0.67 (0.99)	1.22 (0.99)	1.19 (1.22)
Affective	0.99 (0.72)	0.97 (0.64)	0.96 (0.73)	1.08 (0.72)
Behavioral	0.84 (0.78)	0.78 (0.77)	0.81 (0.73)	0.92 (0.75)
Personal impact on planet	1.24 (1.16)	2.81 (0.87)	1.50 (1.44)	3.02 (0.86)
Secondary outcomes				
Intolerance to distress	1.72 (0.89)	1.54 (0.82)	1.65 (0.93)	1.62 (0.82)
Anxiety	1.19 (0.61)	1.20 (0.61)	1.06 (0.58)	1.17 (0.55)
Depression	0.93 (0.64)	0.91 (0.58)	0.96 (0.66)	1.01 (0.70)
Hope	2.77 (0.89)	2.99 (1.13)	2.79 (1.08)	3.30 (1.13)

Abbreviations: ABI, arts-based intervention; APBI, arts-and-philosophy–based intervention; NA, not applicable.

**Table 2.** Results of Mixed Analyses of Variance for Primary and Secondary Outcomes

Outcomes and time^a^	Overall model			
	*F*	*df*	*P* value	Partial η^2^^b^
Primary outcomes				
Global eco-anxiety				
Time (preintervention vs postintervention)	13.73	1234	<.001	0.05
Time × group condition	4.65	1234	.03^c^	0.02
Rumination				
Time	6.69	1230	.01	0.029
Time × group condition	1.06	1230	.30	0.005
Affective				
Time	0.10	1230	.74	0
Time × group condition	1.89	1230	.17	0.008
Behavioral				
Time	0.10	1230	.74	0
Time × group condition	2.46	1230	.11	0.01
Personal impact on planet				
Time	270.92	1232	<.001	0.55
Time × group condition	0.85	1232	.35	0.004
Secondary outcomes				
Intolerance to distress				
Time	7.85	1235	.006	0.03
Time × group condition	1.88	1235	.17	0.008
Anxiety				
Time	1.31	1236	.25	0.006
Time × group condition	1.43	1236	.23	0.006
Depression				
Time	0.004	1233	.94	0
Time × group condition	0.60	1233	.43	0.003
Hope				
Time	10.71	1220	.001	0.04
Time × group condition	2.05	1220	.15	0.04

^a^ Time refers to the passage of time between preintervention and postintervention test measures.

^b^ η^2^ = .01 indicates a small effect size, η^2^ = .06 indicates a medium effect size, and η^2^ = .14 indicates a large effect size.

^c^ In sensitivity analyses for total eco-anxiety, *t*_65_ = 3.6 and *P* < .001 for the arts-based intervention group, and *t*_159_ = 1.56 and *P* = .12 for the arts-and-philosophy–based intervention group.

Both groups showed significant decreases in intolerance to distress (mean [SD] preintervention vs postintervention scores, 1.65 [0.93] vs 1.62 [0.82] for ABI and 1.72 [0.89] vs 1.54 [0.82] for APBI; *P* = .006; partial η^2^ = 0.032) and increases in hope (mean [SD] preintervention vs postintervention scores, 2.79 [1.08] vs 3.30 [1.13] for ABI and 2.77 [0.89] vs 2.99 [1.13] for APBI; *P* = .001; partial η^2^ = 0.046). No differences were found for other outcomes.

## Discussion

This comparative effectiveness research study found that there was an increase in global eco-anxiety in the full sample and that the interaction was a trend. Sensitivity analyses suggest that this increase was more pronounced in the ABI group. Thus, adding philosophical inquiry may serve as a protective factor against some manifestations of eco-anxiety, compared with engaging in artistic creation alone.

All children reported substantial concerns about their personal impact on the planet, suggesting a heightened awareness of anthropogenic effects on the environment.^2^ This awareness could either lead to higher social engagement or to children feeling overburdened with a sense of responsibility for the fate of the planet.^4^ Significant decreases in ruminative eco-anxiety and intolerance to distress nonetheless show promise in supporting children’s mental health, making them feel more confident that the situation can be managed.^5^

These results echo previous work on radical hope,^6^ which acknowledges climate change’s negative impacts and the distress it may bring, while believing in the possibility of a better future.^6^ It involves recognizing the despair brought by climate change, verbalized by children in this study, while working toward improving the planet’s condition.^3^ Fostering radical hope in children may thus involve making place for exploring despair about the future, while being committed to exploring possible solutions to improve our collective fate.

Caution is warranted in how awareness is raised about the importance of social action with children. This may add to the weight some already carry on their shoulders, placing them at a heightened risk of guilt and activist burnout^3^. Children must understand that no individual alone can solve the climate crisis^4^ ; collective action is necessary to dismantle structures perpetuating environmental destruction. Adults should support children in this reflection, while watching for signs of guilt and despair.

The lack of a nontreatment control condition, and the use of 1-item scales to measure eco-anxiety and hope constitute limitations of this study and restrict our conclusions. Future work should address these limitations and focus on scalability of the interventions.

## Notes

Supplement 1. **eAppendix.** Background
eMethods.

eReferences

Supplement 2.
Data Sharing Statement
