# Supplementary material for: Climate Change–Based Art and Philosophy Intervention and Mental Health in Children
Source: JAMA Netw Open. 2025 Sep 11;8(9):e2531298. doi: 10.1001/jamanetworkopen.2025.31298 (PMC12426790; doi:10.1001/jamanetworkopen.2025.31298)
Supplement: Supplement 1. — eAppendix. Background eMethods. eReferences [file jamanetwopen-e2531298-s001.pdf]

Supplemental Online Content

Malboeuf-Hurtubise C, Léger-Goodes T, Dave H, et al. Climate change–based art and philosophy intervention and mental health in children. *JAMA Netw Open.* 2025;8(9):e2531298. doi:10.1001/jamanetworkopen.2025.31298

eAppendix. Background

eMethods

eReferences

© 2025 Malboeuf-Hurtubise C et al. *JAMA Network Open*.

eAppendix. Background

Eco-anxiety refers to direct and indirect impacts of the climate crisis on a range of emotions[1], and youth have been shown to be especially vulnerable to these impacts[2-5]. Such emotional reactions can be accompanied by additional emotions of anger, sadness, despair, fear and guilt, often driven by a strong sense of urgency to act against climate change[6-9]. A notable effect of climate change on youth's mental health encompasses psychological distress that stems from growing up in uncertain times, yet minimal research to date has specifically focused on children's responses to such stressors[10-11]. Early evidence suggests that this distress is associated with children developing feelings of hopelessness, along with a perceived loss of control over their future[12]. In response to this distress, clinicians and researchers in psychology and psychiatry have developed and implemented interventions to promote healthier coping strategies in the context of climate change and eco-anxiety [13–17]. Learning emotional regulation skills, such as emotional expression and voicing concerns, could prevent distress related to climate change[18,19]. Some of these programs have been purposefully designed to be implemented within school settings[20,21] and added to climate change education curriculum, with the intention of promoting coping and children's adaptation [22,23].

Art-based and philosophical activities can be used for personal exploration and a collective construction of meaning through group discussions[24,25]. Artistic creation, for instance, has been shown to foster youth's mental health for more general difficulties, by providing an alternative means to communicate emotions and thoughts, particularly in therapeutic contexts[26–28]. However, artistic activities alone may not always allow participants sufficient time or opportunities to verbalize their reflections on certain issues. Eco-anxiety can elicit fundamental existential concerns, confronting children with difficult questions around identity, death, meaning, and happiness[29,30]. Philosophical inquiry has been shown to help children reflect by engaging in critical

© 2025 Malboeuf-Hurtubise C et al. *JAMA Network Open*.

reflection and group discussions, thereby enabling them to better understand and process sensitive topics and existential questions, such as those related to the climate crisis[10,18,31,32]. Interventions that combine these complementary approaches may offer children a holistic way to process complex issues by blending verbal and non-verbal outlets for introspection, emotional exploration, and expression – especially within school settings[33,34]. However, there is a paucity of research on program development to help children cope with eco-anxiety and the reality of climate change. Indeed, the existing research mainly focuses on climate education and tends to omit the emotional wellbeing of children as they learn about climate change.

eMethods

Hypotheses

We hypothesized that the children assigned to the combined APBI (thus benefitting from opportunities for additional philosophical discussion) would show reductions in self-reported eco-anxiety (primary outcome; the eco-anxiety global score and including the four domains of ruminative, affective and behavioral eco-anxiety, and personal impact on the planet) when compared to children assigned to the ABI, which served as a comparison condition. Similar results were expected for the secondary outcomes of intolerance to distress, anxiety and depression symptoms, and increased hope for the future in the context of climate change.

Design and participants

Students from 14 classrooms in grades three to six, across four public primary schools in England took part in this study and filled out pre-and-post intervention measures. Attrition was minimal in this study: 15 students filled out pre-intervention measures but were absent at post-

© 2025 Malboeuf-Hurtubise C et al. *JAMA Network Open*.

intervention. Classrooms were randomly allocated to the ABI (5 groups; 69 students; 47.7% girls; M_{age} = 9.43 years old) or APBI (8 groups, 169 students; 58.5% girls; M_{age} = 9.84 years old) interventions, using a random numbers table. Data collection took place in the months of June and July 2024.

Procedure and interventions

Both groups received an intervention that was delivered twice a week, for four weeks (total sessions = 8), during school time. Each intervention involved an art activity with ABPI groups having an additional corresponding philosophical discussion: (1) an ugly drawing and discussion on standards of ugliness and beauty (examples of philosophical questions: What makes something beautiful or ugly? Does art need to be beautiful to be considered art?); (2) an emotion wheel and discussion on the meaning of experiencing and expressing emotions (e.g., Why do we have emotions? How is it different to express emotions with words and with art?); (3) a drawing that envisioned the earth in 50 years and discussion on hope and despair for the future state of the planet (e.g., Do you have hope in the context of climate change? Can we have empathy for ourselves when we feel despair?); (4) a photovoice activity on the beauty of nature and discussion on how it can be beautiful (e.g., How do we determine if something is beautiful? Does being in nature feel good because it's beautiful?); (5) a modeling clay and Lego™ activity depicting the strength of nature and discussion on how it can be scary (e.g., What's impressive in nature? How can this power/strength be frightening?); (6) a rock painting activity and discussion on how we can take care of nature (e.g., How can we take care of nature in the context of climate change? What do we need to be able to take care of others and nature?); (7) a photovoice activity on climate change and discussion on our responsibility for nature (e.g., How do you react to a picture that isn't beautiful? Are we responsible for the state of the planet? Do children have the same responsibility as adults

© 2025 Malboeuf-Hurtubise C et al. *JAMA Network Open*.

for the state of the planet?); and (8) a climate slogans activity and discussion on the meaning of change (e.g., What is change? Can we resist change? Can change be frightening?).

With the support of their classroom teacher, the participants completed a questionnaire targeting their eco-anxiety and distress intolerance at pre-test, one week before the beginning of the interventions, and immediately at post-test, one week after the end of the interventions. Randomization occurred after the completion of pre-test measures. Data collection took place during class time and research assistants helped participants to fill out their questionnaire, reading all items out loud and answering questions as needed.

Measures

Primary outcome

The Hogg Eco-Anxiety Scale[35] includes a global scale score of eco-anxiety (9 items; $\alpha_{pre/post}$ = .84) and four subscales, namely affective symptoms (4 items, α_{pre} = .78; α_{post} = .74; e.g. “Feeling nervous, anxious or on edge.”), rumination (1 item, “Unable to stop thinking about losses to the environment”), behavioral symptoms (3 items, α_{pre} = .68; α_{post} = .73; e.g. “Difficulty sleeping”) and anxiety about personal impact (1 item, “Feeling anxious that your personal behaviours will do little to help fix the problem.”). In the context of this study, scores for the global scale and subscales were analyzed.

Secondary outcomes

Participants completed the Distress Intolerance Index for Youth[36] (9 items, α_{pre} = .85; α_{post} = .83; e.g., “Feeling upset scares me), with higher scores measuring higher intolerance to distress. Selected items from the anxiety (3 items, α_{pre} = .62; α_{post} = .63; e.g., “I worry about little things”) and depression subscales (5 items, α_{pre} = .65; α_{post} = .61; e.g., “I feel depressed) of the Behavior

© 2025 Malboeuf-Hurtubise C et al. *JAMA Network Open*.

Assessment System for Children-III[37] were also completed. Finally, children's hope about the future in the context of climate change was assessed using a single item ("Thinking about climate change, how hopeful would you say you are that things will get better in the future?"), on a 4-point Likert scale from "not at all hopeful" to "very hopeful".

Statistical analyses

Descriptive statistics are presented separately for each condition considering pre-to-post-intervention scores. Quantile-quantile plots were used to assess normality. All variables were normally distributed and assumptions for the statistical analyses were met. Please refer to Table 1 for means and standard deviations of pre-to-post scores across groups.

eReferences

1. Doherty TJ, Clayton S. The psychological impacts of global climate change. *Am Psychol*. 2011;66(4):265-276. doi:10.1037/a0023141
2. Léger-Goodes T, Malboeuf-Hurtubise C, Mastine T, Généreux M, Paradis PO, Camden C. Eco-anxiety in children: A scoping review of the mental health impacts of the awareness of climate change. *Front Psychol*. 2022;13. doi:https://doi.org/10.3389/fpsyg.2022.872544
3. Martin G, Reilly K, Everitt H, Gilliland JA. Review: The impact of climate change awareness on children's mental well-being and negative emotions – a scoping review. *Child Adolesc Ment Health*. 2022;27(1):59-72. doi:10.1111/camh.12525
4. Burke SEL, Sanson AV, Van Hoorn J. The Psychological Effects of Climate Change on Children. *Child Fam Disaster Psychiatry*. 2018;20(5):35. doi:10.1007/s11920-018-0896-9
5. Mallet P. The Development of Eco-Anxiety through Middle Childhood and Adolescence. *Psychology*. 2024;15(11):1697-1709. doi:10.4236/psych.2024.1511099
6. Léger-Goodes T, Malboeuf-Hurtubise C, Hurtubise K, et al. How children make sense of climate change: A descriptive qualitative study of eco-anxiety in parent-child dyads. *PLOS ONE*. 2023;18(4). doi:10.1371/journal.pone.0284774
7. Strife SJ. Children's Environmental Concerns: Expressing Ecophobia. *J Environ Educ*. 2012;43(1):37-54. doi:10.1080/00958964.2011.602131

© 2025 Malboeuf-Hurtubise C et al. *JAMA Network Open*.

8. Ojala M. How do children, adolescents, and young adults relate to climate change? Implications for developmental psychology. *Eur J Dev Psychol*. 2023;20(6):929-943. doi:10.1080/17405629.2022.2108396

9. Coffey Y, Bhullar N, Durkin J, Islam MS, Usher K. Understanding Eco-anxiety: A Systematic Scoping Review of Current Literature and Identified Knowledge Gaps. *J Clim Change Health*. 2021;3:100047. doi:10.1016/j.joclim.2021.100047

10. Malboeuf-Hurtubise C, Lefrançois D, Éthier MA, Smith J, Léger-Goodes T, Herba CM. Exploring children's despair in the face of climate change. *Commun Psychol*. 2024;2(1):1-3. doi:10.1038/s44271-024-00130-4

11. Brophy H, Olson J, Paul P. Eco-anxiety in youth: An integrative literature review. *Int J Ment Health Nurs*. 2023;32(3):633-661. doi:10.1111/inm.13099

12. Hickman C. Eco-Anxiety in Children and Young People – A Rational Response, Irreconcilable Despair, or Both? *Psychoanal Study Child*. 2024;77(1):356-368. doi:10.1080/00797308.2023.2287381

13. Herrick IR, Lawson MA, Matewos AM. Through the eyes of a child: exploring and engaging elementary students' climate conceptions through photovoice. *Educ Dev Psychol*. 2022;0(0):1-16. doi:10.1080/20590776.2021.2004862

14. Baudon P, Jachens L. A Scoping Review of Interventions for the Treatment of Eco-Anxiety. *Int J Environ Res Public Health*. 2021;18(18):9636. doi:10.3390/ijerph18189636

15. Bingley WJ, Tran A, Boyd CP, et al. A multiple needs framework for climate change anxiety interventions. *Am Psychol*. 2022;77(7):812. doi:10.1037/amp0001012

16. Birch R. Discerning Hope: Intra-Actions of a Philosophy for Children Workshop and the Eco-Socially Just Potential of Practising Hope. *J Philos Educ*. 2020;54(4):975-987. doi:10.1111/1467-9752.12484

17. Orrù L, Taccini F, Mannarini S. Worry about the Future in the Climate Change Emergency: A Mediation Analysis of the Role of Eco-Anxiety and Emotion Regulation. *Behav Sci*. 2024;14(3):255. doi:10.3390/bs14030255

18. Kankawale SM, Niedzwiedz CL. Eco-anxiety among Children and Young People: Systematic Review of Social, Political, and Geographical determinants. Published online December 19, 2023. doi:10.1101/2023.12.19.23300198

19. Marks E, Atkins E, Garrett JK, et al. Stories of hope created together: A pilot, school-based workshop for sharing eco-emotions and creating an actively hopeful vision of the future. *Front Psychol*. 2023;13. Accessed December 20, 2023. https://www.frontiersin.org/articles/10.3389/fpsyg.2022.1076322

20. Kerret D, Orkibi H, Bukchin S, Ronen T. Two for one: achieving both pro-environmental behavior and subjective well-being by implementing environmental-hope-enhancing programs in schools. *J Environ Educ*. 2020;51(6):434-448. doi:10.1080/00958964.2020.1765131

© 2025 Malboeuf-Hurtubise C et al. *JAMA Network Open*.

21. Ramirez L, Levy SR, Barbosa S, et al. A call for greater collaborative role from psychology in climate change education interventions focused on children/youth. *Can Psychol Psychol Can*. Published online March 27, 2025. doi:10.1037/cap0000408

22. Houghton S, Garvey J, Conor L, et al. Towards an interdisciplinary agenda for teaching in the climate crisis: reflections from the humanities and social sciences. *Environ Educ Res*. 2024;30(11):2007-2019. doi:10.1080/13504622.2023.2273791

23. Chawla L. Childhood Experiences Associated with Care for the Natural World: A Theoretical Framework for Empirical Results. *Child Youth Environ*. 2007;17(4):144-170.

24. Bleazby J, Thornton S, Burgh G, Graham M. Responding to climate change 'controversy' in schools: Philosophy for Children, place-responsive pedagogies & Critical Indigenous Pedagogy. *Educ Philos Theory*. 2023;55(10):1096-1108. doi:10.1080/00131857.2022.2132933

25. Moula Z, Powell J, Karkou V. Qualitative and Arts-Based Evidence from Children Participating in a Pilot Randomised Controlled Study of School-Based Arts Therapies. *Children*. 2022;9(6):890. doi:10.3390/children9060890

26. Bosgraaf L, Spreen M, Pattiselanno K, Hooren S van. Art Therapy for Psychosocial Problems in Children and Adolescents: A Systematic Narrative Review on Art Therapeutic Means and Forms of Expression, Therapist Behavior, and Supposed Mechanisms of Change. *Front Psychol*. 2020;11. Accessed May 13, 2023. https://www.frontiersin.org/articles/10.3389/fpsyg.2020.584685

27. Fancourt D, Garnett C, Spiro N, West R, Müllensiefen D. How do artistic creative activities regulate our emotions? Validation of the Emotion Regulation Strategies for Artistic Creative Activities Scale (ERS-ACA). *PLOS ONE*. 2019;14(2):e0211362. doi:10.1371/journal.pone.0211362

28. Cohen-Yatziv L, Regev D. The effectiveness and contribution of art therapy work with children in 2018 -what progress has been made so far? A systematic review. *Int J Art Ther*. 2019;24(3):100-112. doi:10.1080/17454832.2019.1574845

29. Passmore HA, Lutz PK, Howell AJ. Eco-Anxiety: A Cascade of Fundamental Existential Anxieties. *J Constr Psychol*. 2023;36(2):138-153. doi:10.1080/10720537.2022.2068706

30. Rehling JT. Conceptualising eco-anxiety using an existential framework. *South Afr J Psychol*. 2022;52(4):472-485. doi:10.1177/00812463221130898

31. Giménez-Dasí M, Quintanilla L, Daniel M. Improving emotion comprehension and social skills in early childhood through philosophy for children. *Child Philos*. 2013;9(17):63-89.

32. Lam S, Trott CD. Children's climate change meaning-making through photovoice: Empowering children to learn, care, and act through participatory process. *Educ Soc Cult*. 2022;(62). doi:10.24840/esc.vi62.478

33. Léger-Goodes T, Herba CM, Moula Z, et al. Feasibility, acceptability, and perceived benefits of a creative arts intervention for elementary school children living with speech, language and communication disorders. *Front Child Adolesc Psychiatry*. 2024;3. doi:10.3389/frcha.2024.1322860

© 2025 Malboeuf-Hurtubise C et al. *JAMA Network Open*.

34. Malboeuf-Hurtubise C, Léger-Goodes T, Herba CM, Bélanger N, Smith J, Marks E. Meaning making and fostering radical hope: applying positive psychology to eco-anxiety research in youth. *Front Child Adolesc Psychiatry*. 2024;3. Accessed March 5, 2024. https://www.frontiersin.org/articles/10.3389/frcha.2024.1296446

35. Hogg TL, Stanley SK, O'Brien LV, Wilson MS, Watsford CR. The Hogg Eco-Anxiety Scale: Development and validation of a multidimensional scale. *Glob Environ Change*. 2021;71:102391. doi:10.1016/j.gloenvcha.2021.102391

36. Keller AE, Langer DA, Pincus DB, Meredith Elkins R, Kerns CE, Comer JS. A Psychometric Evaluation of the Distress Intolerance Index for Youth. *J Psychopathol Behav Assess*. 2019;41(3):447-455. doi:10.1007/s10862-018-9711-5

37. Reynolds CR, Kamphaus RW. Behaviour Assessment System for Children – Third Edition Manual. *Am Guid Serv*. Published online 2015.

© 2025 Malboeuf-Hurtubise C et al. *JAMA Network Open*.
