# Supplementary material for: Climate Change–Based Art and Philosophy Intervention and Mental Health in Children
Source: JAMA Netw Open. 2025 Sep 11;8(9):e2531298. doi: 10.1001/jamanetworkopen.2025.31298 (PMC12426790; doi:10.1001/jamanetworkopen.2025.31298)
Supplement: Supplement 2. — Data Sharing Statement [file jamanetwopen-e2531298-s002.pdf]

Data Sharing Statement

Malboeuf-Hurtubise. Climate Change–Based Art and Philosophy and Mental Health in Children. *JAMA Netw Open*. Published September 11, 2025. doi:10.1001/jamanetworkopen.2025.31298

Data
Data available: Yes
Data types: Other (please specify)
Additional Information: Data will be available upon reasonable request to the corresponding author.
How to access data: Data will be available upon reasonable request to the corresponding author.
When available: With publication

Supporting Documents
Document types: None

Additional Information
Who can access the data: Data will be available upon reasonable request to the corresponding author.
Types of analyses: For any purpose
Mechanisms of data availability: With investigator support
